# P-1694. Impact of the BioFire Blood Culture Identification 2 Panel on the Treatment of Bloodstream Infections due to Enterobacterales

**DOI:** 10.1093/ofid/ofaf695.1868

**Published:** 2026-01-11

**Authors:** Lucy Li, Wesley Rogers, Xiaoyue Ma, Harjot K Singh, Jamie Marino, Evan Sholle, Lars Westblade, Michael J Satlin

**Affiliations:** Weill Cornell Medical Center, NYC, NY; NYU Grossman School of Medicine, New York, New York; Weill Cornell Medicine, New York, New York; Weill Cornell Medicine, New York, New York; Weill Cornell Medicine, New York, New York; Weill Cornell Medicine, New York, New York; Weill Cornell Medicine, New York, New York; Weill Cornell Medicine, New York, New York

## Abstract

**Background:**

Rising rates of extended-spectrum β-lactamase-producing *Enterobacterales* (ESBL-Es) pose challenges for clinicians selecting antimicrobial agents for bloodstream infections (BSIs) before antimicrobial susceptibility data are available, where the need to choose effective therapies is balanced against avoiding unnecessary broad-spectrum therapies. We aimed to determine if the BIOFIRE Blood Culture Identification 2 (BCID2) panel (bioMérieux), which detects the most common ESBL gene (*bla*_CTX-M_*)* from positive blood cultures, decreases time to effective therapy in ESBL-E BSIs and time to antimicrobial de-escalation in non ESBL-E BSIs.

**Figure:**
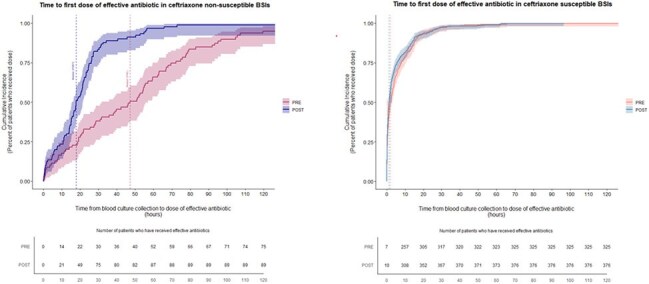
The cumulative incidence curves for the PRE-BCID2 cohort vs the POST-BCID2 cohort are shown for ceftriaxone-non-susceptible and ceftriaxone-susceptible patients respectively. The time to the first dose was defined as the interval between time of blood culture collection and time that the first dose of an effective antibiotic was administered. Effective antibiotics were defined based on institutional and Infectious Disease Society of America guidelines and susceptibility results. At 120 hours, the number of patients in the table does not equal the total number of patients in the sub-cohort as some patients never received effective therapy. Abbreviations: BCID2, Biofire Blood Culture Identification Panel 2; BSI: bloodstream infection

**Figure:**
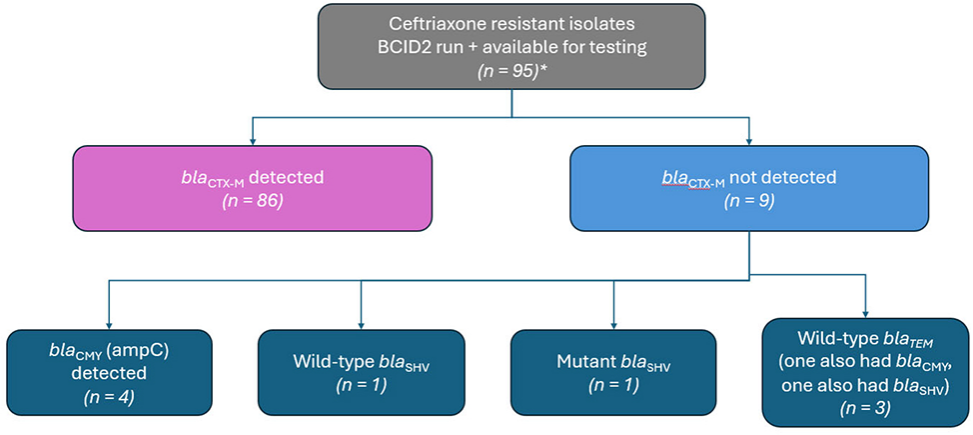
*Patients were excluded from overall analysis if they never received a dose of antibiotic at one of our institutions, so 92 were analyzed for outcomes. Isolates obtained after 01/2022 were available for testing with an expanded antimicrobial resistance panel. Out of the 95 with a BCID2 assay result, 86 had blaCTX-M detected by the expanded panel. Of the remaining nine, blaCMY alone was detected in four, blaSHV alone was detected in two (one mutant, one wild-type), and blaTEM was detected in three. Of the three isolates harboring blaTEM, one also harbored blaCMY and one also harbored blaSHV. Abbreviations: BCID2, Biofire Blood Culture Identification Panel 2

**Methods:**

Data from an academic center and a community hospital were extracted from the electronic health record using Structured Query Language (SQL) queries against a relational database. We compared BSIs due to *Escherichia coli*, *Klebsiella oxytoca*, and *Klebsiella pneumoniae* during the year before and after the BCID panel (which does not detect *bla*_CTX-M_) was replaced by the BCID2 panel in August 2022. The primary outcome for BSIs due to ceftriaxone-non-susceptible (CRO-NS) isolates was time to effective therapy (carbapenem or active non-β-lactam agent). Cumulative incidence plots were generated using the method of Fine and Gray to account for competing events. The primary outcome for BSIs due to CRO-susceptible (CRO-S) isolates was time to de-escalation from an anti-pseudomonal β-lactam agent to a more narrow-spectrum agent. We also assessed β-lactamase genes in CRO-NS isolates.

**Table 1:**
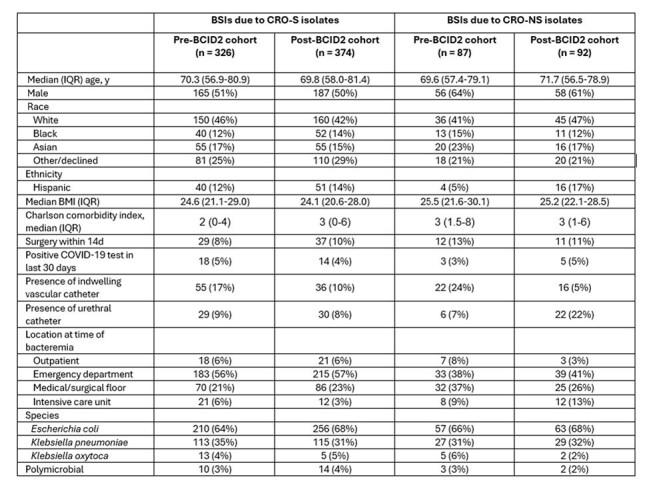
Demographic and Baseline Clinical Characteristics Abbreviations: BCID2, BioFire Blood Culture Identification Panel 2; BMI, body mass index; BSI: bloodstream infection; CRO-S, ceftriaxone-susceptible; CRO-NS: ceftriaxone-nonsusceptible

**Table 2:**
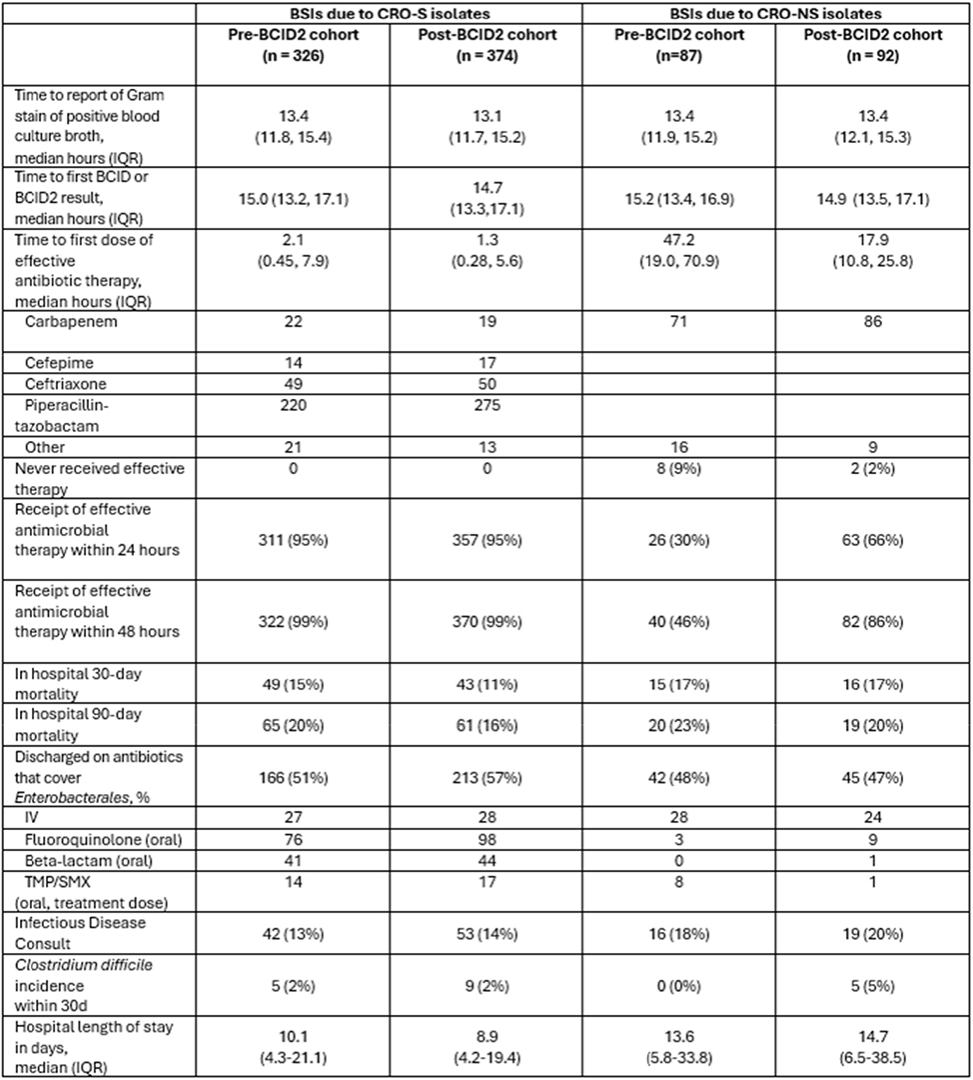
Clinical and Microbiologic Outcomes Abbreviations: BCID2, BioFire Blood Culture Identification Panel 2; BMI, body mass index; BSI: bloodstream infection; CRO-S, ceftriaxone-susceptible; CRO-NS: ceftriaxone-non-susceptible; TMP/SMX, trimethoprim/sulfamethoxazole * For all measures, t=0 was defined as the time of blood culture collection

**Results:**

Median time to effective therapy was reduced from 47.2 hours (IQR 19.0-70.9; n = 87) to 17.9 hours (IQR 10.8-25.8; n = 92) for CRO-NS BSIs after BCID2 implementation. Median time to de-escalation in CRO-S BSIs was 62.2 hours (IQR 45.7-90,3; n = 326) in the pre-BCID2 cohort and 67.0 hours (IQR 49.9-98.0; n = 92) in the post-BCID2 cohort. Of 95 CRO-NS isolates available for molecular testing, 86 harbored *bla*_CTX-M_ and 9 harbored other genes.

**Conclusion:**

Rapid detection of CTX-M via BCID2 implementation was associated with a reduction in time to effective therapy for BSIs due to CRO-NS isolates, but not a reduction in time to de-escalation for BSIs due to CRO-S-isolates. CTX-M was the mechanism of CRO resistance in 91% of CRO-NS isolates.

**Disclosures:**

Lars Westblade, PhD, Elements Materials Technology: Grant/Research Support|Hardy Diagnostics: Grant/Research Support|Melinta Therapeutics: Grant/Research Support|Selux Diagnostics: Grant/Research Support|Shionogi: Advisor/Consultant|SNIPRBIOME: Grant/Research Support Michael J. Satlin, MD, MS, AbbVie: DSMB participant|bioMerieux: Grant/Research Support|Merck: Grant/Research Support|SNIPRBiome: Grant/Research Support

